# GeNePi: a graphics processing unit enhanced next-generation bioinformatics pipeline for whole-genome sequencing analysis

**DOI:** 10.1093/bib/bbag001

**Published:** 2026-01-25

**Authors:** Stefano Marangoni, Federica Furia, Debora Charrance, Agata Fant, Salvatore Di Dio, Sara Trova, Giovanni Spirito, Francesco Musacchia, Alessandro Coppe, Stefano Gustincich, Manuela Vecchi, Fabio Landuzzi, Andrea Cavalli

**Affiliations:** Computational and Chemical Biology, Italian Institute of Technology (IIT), CMP3VdA, Via Lavoratori - Vittime del Col du Mont 28, 11100 Aosta, Italy; Computational and Chemical Biology, Italian Institute of Technology (IIT), Center for Clinical and Computational Genomics, Via Lavoratori - Vittime del Col du Mont 28, 11100 Aosta, Italy; Computational and Chemical Biology, Italian Institute of Technology (IIT), CMP3VdA, Via Lavoratori - Vittime del Col du Mont 28, 11100 Aosta, Italy; Computational and Chemical Biology, Italian Institute of Technology (IIT), Center for Clinical and Computational Genomics, Via Lavoratori - Vittime del Col du Mont 28, 11100 Aosta, Italy; Computational and Chemical Biology, Italian Institute of Technology (IIT), CMP3VdA, Via Lavoratori - Vittime del Col du Mont 28, 11100 Aosta, Italy; Computational and Chemical Biology, Italian Institute of Technology (IIT), Center for Clinical and Computational Genomics, Via Lavoratori - Vittime del Col du Mont 28, 11100 Aosta, Italy; Non-coding RNAs and RNA-based therapeutics, Italian Institute of Technology (IIT), CMP3VdA, Via Lavoratori - Vittime del Col du Mont 28, 11100 Aosta, Italy; Computational and Chemical Biology, Italian Institute of Technology (IIT), CMP3VdA, Via Lavoratori - Vittime del Col du Mont 28, 11100 Aosta, Italy; Computational and Chemical Biology, Italian Institute of Technology (IIT), Center for Clinical and Computational Genomics, Via Lavoratori - Vittime del Col du Mont 28, 11100 Aosta, Italy; Non-coding RNAs and RNA-based therapeutics, Italian Institute of Technology (IIT), CMP3VdA, Via Lavoratori - Vittime del Col du Mont 28, 11100 Aosta, Italy; Non-coding RNAs and RNA-based therapeutics, Italian Institute of Technology (IIT), Center for Clinical and Computational Genomics, Via Lavoratori - Vittime del Col du Mont 28, 11100 Aosta, Italy; Non-coding RNAs and RNA-based therapeutics, Italian Institute of Technology (IIT), CMP3VdA, Via Lavoratori - Vittime del Col du Mont 28, 11100 Aosta, Italy; Non-coding RNAs and RNA-based therapeutics, Italian Institute of Technology (IIT), Center for Clinical and Computational Genomics, Via Lavoratori - Vittime del Col du Mont 28, 11100 Aosta, Italy; Non-coding RNAs and RNA-based therapeutics, Italian Institute of Technology (IIT), CMP3VdA, Via Lavoratori - Vittime del Col du Mont 28, 11100 Aosta, Italy; Non-coding RNAs and RNA-based therapeutics, Italian Institute of Technology (IIT), Center for Clinical and Computational Genomics, Via Lavoratori - Vittime del Col du Mont 28, 11100 Aosta, Italy; Computational and Chemical Biology, Italian Institute of Technology (IIT), CMP3VdA, Via Lavoratori - Vittime del Col du Mont 28, 11100 Aosta, Italy; Computational and Chemical Biology, Italian Institute of Technology (IIT), Center for Clinical and Computational Genomics, Via Lavoratori - Vittime del Col du Mont 28, 11100 Aosta, Italy; Non-coding RNAs and RNA-based therapeutics, Italian Institute of Technology (IIT), CMP3VdA, Via Lavoratori - Vittime del Col du Mont 28, 11100 Aosta, Italy; Non-coding RNAs and RNA-based therapeutics, Italian Institute of Technology (IIT), Center for Clinical and Computational Genomics, Via Lavoratori - Vittime del Col du Mont 28, 11100 Aosta, Italy; Non-coding RNAs and RNA-based therapeutics, Italian Institute of Technology (IIT), Center for Human Technology, Via Morego 30, 16152 Genova, Italy; Non-coding RNAs and RNA-based therapeutics, Italian Institute of Technology (IIT), CMP3VdA, Via Lavoratori - Vittime del Col du Mont 28, 11100 Aosta, Italy; Non-coding RNAs and RNA-based therapeutics, Italian Institute of Technology (IIT), Center for Clinical and Computational Genomics, Via Lavoratori - Vittime del Col du Mont 28, 11100 Aosta, Italy; Computational and Chemical Biology, Italian Institute of Technology (IIT), CMP3VdA, Via Lavoratori - Vittime del Col du Mont 28, 11100 Aosta, Italy; Computational and Chemical Biology, Italian Institute of Technology (IIT), Center for Clinical and Computational Genomics, Via Lavoratori - Vittime del Col du Mont 28, 11100 Aosta, Italy; Computational and Chemical Biology, Italian Institute of Technology (IIT), CMP3VdA, Via Lavoratori - Vittime del Col du Mont 28, 11100 Aosta, Italy; Computational and Chemical Biology, Italian Institute of Technology (IIT), Center for Clinical and Computational Genomics, Via Lavoratori - Vittime del Col du Mont 28, 11100 Aosta, Italy; Computational and Chemical Biology, Italian Institute of Technology (IIT), Center for Human Technology, Via Morego 30, 16152 Genova, Italy; Centre Européen de Calcul Atomique et Moléculaire (CECAM), Ecole Polytechnique Fédérale de Lausanne, 1015 Lousanne, Switzerland

**Keywords:** bioinformatics pipeline, genomic variants, whole-genome sequencing analyses, next-generation sequencing, GPU-accelerated algorithm, Nvidia Clara Parabricks, Nextflow

## Abstract

Next-generation sequencing (NGS) has revolutionized genome biology by enabling rapid whole-genome sequencing (WGS) and driving its adoption in research and clinical settings. However, the high-throughput nature of NGS and the complexity of downstream analyses demand robust computational solutions. We present GeNePi, a modular bioinformatic pipeline for efficient and accurate analysis of WGS short paired-end reads. GeNePi is a genomics analysis pipeline built on the Nextflow framework, integrating graphics processing unit (GPU)-accelerated algorithms from NVIDIA Clara Parabricks to enable high-performance variant discovery. The pipeline supports multiple workflow configurations and automates the detection of a broad range of genomic variants, including single-nucleotide variants and small insertions/deletions via GPU-accelerated HaplotypeCaller, copy number variants (CNVs) using CNVkit, and structural variants through a consensus approach combining Manta, Lumpy, BreakDancer, and CNVnator. Additionally, GeNePi incorporates MELT for the detection of mobile element insertions, providing a comprehensive framework for variant discovery and characterization. Benchmarking on synthetic and real datasets demonstrates high accuracy and performance comparable to state-of-the-art tools such as Genome Analysis ToolKit (GATK), establishing GeNePi as a scalable solution for comprehensive WGS analysis. These features make GeNePi a valuable instrument for large-scale analyses in both research and clinical contexts, representing a key step towards the establishment of National Centers for Computational and Technological Medicine.

## Introduction

Next-generation sequencing (NGS) has transformed genome biology by dramatically improving sequencing efficiency and reducing the time required to sequence an entire human genome to <48 h [1]. As NGS technology becomes more accurate and accessible, it is increasingly integrated into research and clinical applications. The *100000 Genome Project* has demonstrated that whole-exome sequencing and whole-genome sequencing (WGS) can now be routinely incorporated into clinical care.

Given the high-throughput nature of NGS technologies, there is a need for concomitant advancements of powerful computational infrastructures and bioinformatic tools to process complex and big genomic data. NGS sequences require various elaborations to be combined into a collection of variants that can be interpreted and prioritized for research and clinical purposes. Standardized data processing pipelines are crucial to ensuring consistency across studies, improving result reproducibility and streamlining researchers’ workflows. Nowadays, pipelines are increasingly integrated as a stable component of the data analysis process, either provided as a service (i.e. Dragen [2]), or community-developed solutions (i.e. Sarek [3]) or by developing in-house frameworks. However, computational challenges remain, including high resource demands, the need for specialized hardware, and long execution times [4].

High-performance computing (HPC) systems are well suited for handling large-scale genomic data while ensuring high levels of data security [5, 6]. The adoption of parallelized algorithms capable of scaling across multi-node systems has become essential. Additionally, the advent of graphics processing units (GPUs) has revolutionized computational sciences by significantly reducing analysis time through parallelization. The genomics field has benefited from GPU acceleration, particularly with the introduction of Nvidia Clara Parabricks that enables efficient analysis of large genomic datasets. GPUs offer distinct advantages over traditional computing methods, including hardware reusability, cost-effectiveness, and the elimination of ongoing subscription fees. Their ability to support multiple analyses further enhances computational efficiency and infrastructure flexibility.

To address these computational challenges, we present GeNePi, an innovative GPU-enhanced Next-Generation bioinformatics pipeline designed for the accurate processing of WGS short paired-end reads. Developed using Nextflow with a modular approach, GeNePi integrates GPU-accelerated algorithms and supports multiple workflow selections. It automates key variant identification steps, including single-nucleotide variants (SNVs), small insertions or deletions (INDELs), copy number variants (CNVs), and structural variants (SVs). By leveraging HPC environments, GeNePi optimizes resource allocation through job scheduling and parallelization, improving efficiency and scalability. Its flexible design allows adaptation to various research and clinical applications, significantly enhancing the speed of large-scale WGS analyses. Overall, GeNePi represents a powerful tool for genomic research, enabling users to focus on biologically relevant insights with minimal computational overhead.

## Materials and methods

### GeNePi description

GeNePi is based on the Nextflow platform and we adopted Singularity (or equivalently Apptainer) for the containerization of the requisite tools. The pipeline was written following a modular approach to facilitate the re-analysis of the samples. Each module is composed of multiple processes that Nextflow submits to an “executor,” empowering the parallelization between independent processes. The resources allocated to each process and the parameters employed by the bioinformatics tools can be adjusted by the users through the configuration file.

#### Alignment and variant calling using graphics processing unit-accelerated algorithms

The module **PB_germ** (see Supplementary S1) performs the alignment, variant calling of the single-nucleotide variants (SNVs) or of the small insertions/deletions (INDELs), and the soft-filtering of the variants. We adopted the *germline_pipeline* from the NVIDIA Clara Parabricks suite (v4.3.0) that involves porting the GATK workflow to GPUs. Subsequently, the VCF files generated during the variant calling are soft-filtered with the software VariantFiltration (GATK v4.3.0.0). A comprehensive list of the parameters is provided in Supplementary S2.1. Notably, low-quality variants are not removed from the VCF at this stage allowing manual inspection by the user.

#### Annotation

The module **germline_snv_annotation** accepts a series of VCF files as input and it incorporates a comprehensive set of information to facilitate variant prioritization and interpretation. The module applies the multi-allelic split (Bcftools v1.21.0) and annotates the resulting VCF with three tools: SnpEff (v5.1) [7] to include information on the putative effect on the transcripts; ANNOVAR (databases updated to 23 January 2024) [8] to include information on the frequency of the variant in the population studies, score predictions, predicted effects of pathogenicity, or clinically observed effects; and optionally COSMIC (Catalogue of Somatic Mutations in Cancer) a database reporting the somatic mutations in human cancer [9] (more details are available on the Supplementary S2.2). This module follows **PB_germline** in the main flux or it may function as an independent workflow by using the entry **SNV_annot** (see Supplementary S1).

#### Single-nucleotide variants and insertions/deletions prioritization

The module **snv_filt** accepts a list of annotated VCF files as an input that are then processed through an in-house pipeline designed to prioritize potentially disease-causing variants. Initially, common polymorphisms in the Genome Aggregation Database (GnomAD) (v4.0) are discarded using the tool SnpSift and imposing a double threshold on the allele frequency (AF $\leq $ 5% and AF $\geq $ 95%) [10]. In the second instance, we searched variants with the potential to impact protein structure and splicing according to SnpEff annotation or with known clinical effects according to Annovar annotation. The third filtration step select variants that meet a minimum quality standard, as defined by the FILTER fields of the VCF. This step is crucial to minimize false positives (FP) in the final VCF. The fourth filtration step is gene-centric, based on the selection of genes associated with specific diseases. The user has the option of defining its own list. The final filtration retains variants that can be defined as highly clinically relevant based on the Annovar annotation. Specifically, the evaluation considers pathogenic or likely pathogenic variants as defined by InterVar [11], while variants of unknown significance undergo additional scrutiny using the same criteria as the ClinVar Clinical Interpretation and Reporting System CLINSIG tier. For further information on the filtration steps, refer to Supplementary S2.2.2. In the event that no disease-causing variants are detected in the initial analysis, re-evaluation may be necessary. To this end, we retained the VCF produced at each step of the filtration process, enabling expeditious retrieval of previously discarded variants.

#### Structural variants consensus

The module **SV_consensus** is a consensus pipeline for the detection of SVs, inspired by a previous workflow described by Vialle *et al.* [12]. It integrates the flown into Nextflow and adopts a consensus with four variant callers, to reduce computational resources and execution time. It can be run independently, using the entry **SV_consensus**, or as part of the default workflow. Briefly, the channel accepts as input a list of BAM files on which SVs calling is performed with: Manta (v1.6.0) [13], Lumpy (v0.2.13) [14], CNVnator (v0.3.3) [15], and BreakDancer (v1.4.5) [16]. As described by Vialle *et al.* [12], a consensus is run using Survivor (v1.0.7) [17] dividing SVs in SV-type groups. The VCF file containing the merged deletions, duplications, and inversions is then genotyped using the Smoove (v0.2.6) [18] *genotype* function. Finally, to enhance the precision of the analysis, we implemented the Duphold method [19] on the deletions and duplications. This approach adds the values DHBFC and DHFFC to the VCF information fieldsthat are used for filtering with the thresholds recommended by the authors. Optionally, we incorporated a functionality to detect mobile element insertions (MEIs) using the tool MELT [20]. This additional analysis is resource-intensive; since it is not typically required, it can be disabled by the user (further details are available in Supplementary S2.2.4).

#### Structural variants annotation and filtration

The modules **sv_annotation** and **sv_filt**, annotate the merged SV calls and apply an in-house filtration to prioritize the SVs, respectively. The **sv_annotation** employes the SVAFotate (v0.0.1) [21] and AnnotSV (v3.2.3) [22] annotations. First SVAFotate incorporates population-level allele frequency information and associated metrics. Subsequently, AnnotSV adds functionally, regulatory, and clinically relevant information. The **sv_filt** includes an in-house script for variants filtration. The variants can be filtered using a list of relevant genes and prioritizing only rare (${\textrm{MAF}} <1\%$) SVs. Haploinsufficiency and triplosensitivity scores are incorporated into the final output for each respective gene in the context of deletions and duplications, as dosage-sensitive genes could be associated with disease [23] (for more details on the filtering script, see Supplementary S2.2.5). The table generated by our pipeline contains the prioritized SVs and is divided into different sheets: *shortCNV* contains deletions and duplications with a length smaller than 500 kb; *longCNV* contains deletions and duplications longer than 500 kb; *INS only in Manta* contains the insertions identified by Manta; *MEI* contains the mobile element insertions; *INV* all the inversions; and *TRA* all the transpositions. The script employs the samplot module [24] to generate plots for the visualization of SVs, that could facilitate the manual curation of the identified SVs. These modules can be executed as an independent workflow, with the option to select the entry **SV_annot_filt** (see Supplementary S1).

#### Copy number alterations

CNVkit [25] is a robust and efficient tool for the detection of copy number alterations (CNAs). We decide to dedicate a module to the analysis of large CNA based on CNVkit. The **CNVkit_wf** module accepts a list of BAM files as an input, and it can be utilized either in conjunction with **PB_germ** workflow or, alternatively, as an independent process by employing the entry **CNVkit_wf**. Initially, the BAMs are processed via the CNVkit *batch* function, and then, the generated output files are processed by an in-house Python script, resulting in the production of chromosomes plots that include information on genes dosage sensitivity [26]. This strategy allows a rapid visualization of large CNAs with possible deleterious effects.

### Benchmarking

#### Synthetic and real next-generation sequencing data

We used three different datasets to validate our pipeline: two synthetic data and the Ashkenazi son HG002 (NA24385) dataset from the Genome in a Bottle (GIaB) Consortium. A first set of synthetic data was generated using wgsim. Based on the hg38 reference genome, we created a pair of pair-end FASTQ files, simulating an average coverage of 100$\times $, and inserting 2 924 993 random SNVs and INDELs to serve as the ground truth for benchmarking purposes. The second synthetic dataset was then generated to evaluate the ability of the pipeline to detect SVs. To this end, we created some specific genomic rearrangements on the Hg38 reference genome using *simuG*, [27]: two translocations were generated on one allele; while on the other, we inserted one inversion and one deletion (see Supplementary S3.3.1 for the precise identification of the SVs position). Then, we combined the two alleles to produce a diploid genome and use the technique adopted in the aforementioned dataset to generate the synthetic reads.

To evaluate the accuracy metrics, the SNVs and INDELs calls were assessed against the ground truth using Hap.py, while the SVs calls were evaluated using Truvari [28]. *Hap.py* was designed by Illumina for the specific purpose of comparing the germline SNVs/INDELs calls by an analysis tool to a truth set. The comparison is conducted based on the position and consistency of the reference and alteration between VCF files. *Truvari*, a toolkit for benchmarking, merging, and annotation of SVs, facilitates a comparison of variants called by an analysis tool to a truth set. This comparison is based on five parameters: the SV type, the distance from the reference, the reciprocal overlap, the similarity of the size and of the sequence, and the genotype. Both tools are available as modules of the nf-core variantbenchmarking pipeline. The “Ashkenazi son” HG002 (NA24385) is a publicly available dataset released by the GIaB consortium hosted by the National Institute of Standards and Technology (NIST). It is one of the genomes belonging to a family trio included in the selected material from the Personal Genome Project [29]. HG002 genome is a widely used dataset for benchmarking, as this sample has been sequenced with several distinct technologies, thereby enabling a comprehensive analysis of the genomic variants present in the sample [30]. In this specific context, the confirmation of various variants of the “Ashkenazi son” HG002 genome enabled the release of datasets that could be utilized for benchmarking analytical tools and SNVs [31] and SVs [32] “truth sets.” The variants were then classified into true positives (TP), FP, or false negatives (FN). Precision, recall, and F1 score were calculated independently for SNPs, INDELs, and SVs, providing a comprehensive assessment of the variant calling performance.

## Results

### Overview of the GeNePi pipeline structure

**Figure 1:**
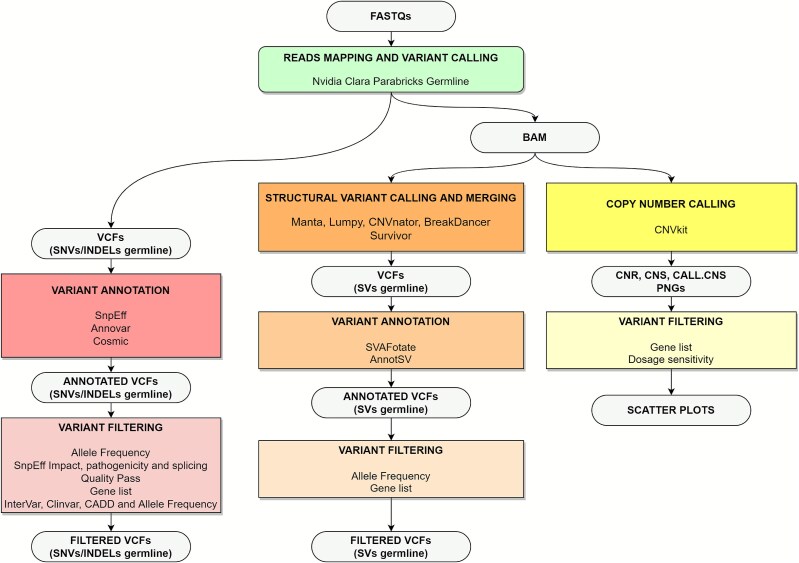
Schematic representation of the analysis pipeline: first, a list of paired FASTQ files is retrieved from the archive and processed through a GPU-accelerated alignment and short-variant calling workflow; then, the resulting data are split into three independent streams: (i) single-nucleotide variant (SNV) annotation and filtering, (ii) structural variant (SV) calling, annotation, and filtering, and (iii) large copy-number variant (CNV) calling and filtering.

The GeNePi pipeline (available at GeNePi) is based on Nextflow and has been developed in the DSL2 language version to be compatible with the latest JAVA version. To ensure portability to and compatibility with HPC infrastructures, the tools are containerized using Apptainer. By default, the pipeline is designed to fully address all steps in the process, from the alignment of pair-end reads, from FASTQ files, to the prioritization of SNVs, CNVs and SVs (see Fig. 1). Depending on the user needs, the pipeline may be configured to follow different workflows and it supports customization via configuration files. These features enable fast re-analysis of specific modules, allowing optimization of resources requirements and better adaptation to user need. The workflow offers flexible entry points, and analyses are initiated independently for each sample. This methodological framework enables trivial parallelization across samples. The download of the required resources is performed during the initial configuration phase. In an HPC environment, this setup is required only once, as the pipeline can be shared among multiple users.

Compared with other available solutions GeNePi and nf-core/Sarek share a modular architecture implemented in Nextflow, enabling reproducible and scalable genomic workflows with user-selectable algorithms for alignment and variant calling. The main distinction lies in the default use of GPU-accelerated tools. GeNePi leverages GPU-parallelized software for alignment and SNV calling, computational bottlenecks in most workflows, whereas the original version of Sarek relies on two CPU-based alternatives, although recently the code employ GPU-parallelized algorithm for the alignment. The two CPU-based alternatives include a Sentieon-powered workflow offering approximately a tenfold speed-up over standard GATK, and a BWA-GATK approach that parallelizes analysis across multiple reference intervals for an estimated threefold improvement, assuming optimal job scheduling. Beyond performance, GeNePi integrates a prioritization module to highlight clinically relevant variants and employs a consensus SV caller to enhance the detection of genomic rearrangements. Although prioritization may exclude common polymorphisms or variants of uncertain significance, the workflow maintains full traceability, allowing users to revisit earlier filtering steps for comprehensive review.

### Graphics processing unit-accelerated paired-end reads alignment and variant calling

GATK, a software widely used by the scientific community, and the Broad Institute guidelines are regarded as the gold standard in the field of genomic analysis [33]. Despite its broad use, the computational time to process WGS data using classic CPU-based tools as GATK is a limiting factor. To overcome this limitation different solutions are available, i.e. the use of: a toolkit that (i) optimizes the process on a multi-core processor as Sentieon [34] or (ii) employs dedicated hardware like the FPGA based Dragen toolkit suite [2] or (iii) the GPU accelerated NVIDIA Clara Parabricks suite. We decided to integrate in our workflow the *germline* pipeline from the NVIDIA Clara Parabricks suite (v4.3.0). This tool is equivalent to the Broad Institute Germline Single-Sample Data, yet, it drastically reduces the execution time compared with CPU-based [35] GATK workflow for germline variant calling. Additionally, its execution time is comparable to that of the FPGA-based Dragen [36].

**Figure 2:**
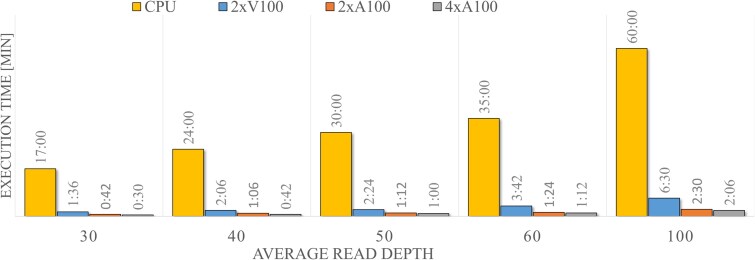
Comparative runtime analysis of NVIDIA Clara Parabricks and CPU-based GATK germline variant-calling workflows: benchmarks were performed across four hardware configurations (2$\times $V100, 2$\times $A100, 4$\times $A100, and CPU; details in Section Materials amd methods) at multiple genome coverage levels (30$\times $, 40$\times $, 50$\times $, 60$\times $, and 100$\times $) showing an almost linear rise in processing time (minutes) with coverage, with the performance gap between CPU-based and GPU-accelerated workflows widening at higher coverage depths.

In our comparative analysis of the Nvidia Clara Parabricks and the equivalent CPU-based GATK workflow, we observed an execution time for the CPU based module of $\sim $17 h for a sample sequenced at 30$\times $, that increased linearly with sequencing depth reaching $\sim $60 h at 100$\times $ coverage (see Supplementary S3.1 for information on the computational infrastructure). Similarly, the execution time of the GPU-accelerated module exhibits a linear dependence on coverage; however, the results demonstrate an average acceleration in execution time ranging from $10$-fold to $78$-fold (see in Fig. 2 and Supplementary Fig. S10), depending on the hardware configuration. These findings indicate that this approach can significantly improve the efficiency of genomic analysis, compared with the conventional GATK-based alignment and variant calling workflow.

We evaluated the germline subworkflow of GeNePi using HG002 WGS data at 60$\times $ coverage across different GPU architectures, GPU allocations, and Clara Parabricks versions to quantify performance improvements. Newer GPU architectures and higher GPU counts significantly reduce runtime (with a good scalability up to $4$ GPUs, see Supplementary Fig. S11). Indeed, allocating additional GPUs does not provide a cost effective computational advantage. We also observed an 30% reduction in runtime when using Clara Parabricks v4.6 compared with v4.3, confirming the impact of ongoing software optimization, although the newer versions has discontinued support for previous GPUs architecture.

### Call and prioritization of single-nucleotide variants and insertions/deletions

Once variants have been identified, it is necessary to collect biological information on the variants. As stated in the ACMG guidelines, the variant annotation process is part of the workflow of the analysis of genomic data. The biological information is then used to prioritize the variants, in order to pinpoint the variants with major relevance [37]. Our annotation module employs three different software, namely SnpEff, Annovar, and Cosmic, to incorporate a comprehensive set of information that could facilitate the prioritization and interpretation of the identified variants. Given the substantial number of variants typically identified in a WGS sample (approximately several millions), we have implemented an automatic, yet programmable, set of filters to prioritize the variants. The filtration scheme is structured in multiple steps with the goal of refining variant selection by progressively excluding those that do not meet increasingly stringent criteria (details in Section Structural variants annotation and filtration in Materials and methods). The filtering approach we designed allowed to reduce the number of SNVs/INDELs variants from $\sim $5 million for a WGS to 4–5 considering the Hereditary Breast and Ovarian Cancer genes panel [38], or to 132 considering a comprehensive list of 900 cancer-related genes (a list that include the Cancer Gene Census [39]). Therefore, limiting the number of candidate variants to a few tens or hundreds significantly decreases the time required for variant interpretation and classification in a clinical setting. The prioritization strategy is designed to efficiently identify variants with potential clinical relevance by focusing on exonic and near-exonic regions and on variants previously associated with disease. This targeted approach reduces noise from common polymorphisms, thereby improving interpretability and diagnostic yield. Importantly, the multi-step workflow incorporates mechanisms that allows users to revisit variants initially deprioritized, ensuring flexibility and completeness in the analysis. Although, this iterative process entails a modest increase in storage requirements, it enhances the robustness of variant assessment and supports comprehensive genomic interpretation.

### Consensus on structural variants callers

Structural variants are defined as genomic rearrangements involving at least 50 bp. These variants encompass a range of structural alterations, including deletions, insertions, duplications, inversions, and translocations. SVs have the potential to affect a significant portion of the genome and have been previously linked to specific cancer types [40, 41] and other diseases [42], including brain-related disorders. Consequently, the accurate detection of SVs is crucial for identifying disease-causing variants. The technical limitations imposed by short-read sequencing technology have hindered the identification of this source of genomic variability [43]. Prevailing algorithms for SVs identification with short pair-end reads are based on the identification of discordant/aberrant pair reads, spliced reads or variation in coverage depth. Among these, the most commonly adopted are Manta [13], Lumpy [14], Breakdancer [16], SvABA [44], and Delly [45]. However, there is no consensus on the optimal tool to adopt. Consequently, the prevailing strategy is to consider a combination of tools, aiming to leverage the advantages of each while mitigating the limitations of a single tool. We performed a comprehensive benchmarking of different available tools and, following a thoroughly evaluation of the results, we adopted a consensus strategy comprising four tools, similar to the approach outlined by Vialle *et al.* [12]. This strategy has been shown to enhance precision and the F1 score when compared with performance of individual tools (see Structural variants consensus).

For an extensive analysis of CNAs, a recurrent form of genetic variations particularly relevant in cancer, we also integrated a module dedicated to the specific identification of CNAs. Based on the software CNVkit, we implemented a script to facilitate the visualization of relevant alterations on a user-defined set of genes that include information on the dosage sensitivity score (see section “Copy number alterations” in Materials and methods).

### Variant detection benchmark

We performed a comprehensive benchmarking analysis to evaluate the performance and accuracy of our pipeline in the detection of SNVs, INDELs, and SVs using the *variant benchmarking* module of nf-core pipeline [46]. This analysis is crucial for comparing the performance of various available tools in terms of sensitivity, specificity, FP, and FN rates. The primary goal of these tests was to evaluate the performances of the tools we developed and ensure that they could produce reliable and consistent results, thereby minimizing errors in variant detection. The benchmarking has been performed on the HPC infrastructure that has been developed for the 5000genome@vda project (further details are available in the Supplementary S3.1).

Initially, we tested our pipeline using two different sets of synthetic data. The first set was designed for identification of SNVs and INDELs, whereas the second set for the detection of various types of SVs including deletions, duplications, insertions, inversions, and translocations. The former set was generated from the reference genome *hg38* using wgsim, a widely used tool for simulating WGS reads. In this file, we inserted $2\,924\,993$ random SNVs/INDELs to serve as the ground truth for the purposes of the benchmarking. The pipeline identifies a total of $3\,044\,123$ variants. Subsequently, we performed the benchmarking analysis using Hap.py.

Detailed values and metrics are provided (see Supplementary Table S3.3.3). Data demonstrated that overall F1, recall and precision values for SNPs detection is >0.964, higher than in INDELs detection. Although the recall and precision of the INDELs detection seems to be lower than expected (Recall: 0.869 considering all calls and 0.573 considering only PASS variants; Precision: 0.641 and 0.957 for ALL and PASS, respectively), a thorough examination of the results reveals that this discrepancy is primarily due to the non-standard coordinate definition in the truth set (see Supplementary S3.3.1).

The second synthetic dataset was generated using simuG [27], a tool designed for simulating various types of SVs, such as deletions, duplications, insertions, inversions, and translocations. These synthetic SVs provided a controlled environment to test the robustness of our pipeline in detecting complex genomic rearrangements. Following the processing of the synthetic data through our pipeline, we confirmed that all the expected SVs were accurately detected, thereby demonstrating the ability of the pipeline to detect different types of SVs (Supplementary Table S3.3.1). Importantly, the pipeline was also able to accurately identify the position of the breakpoints (Supplementary Table S3.3.1). Collectively, these preliminary results on synthetic data suggest that the pipeline accurately detects both SNVs and SVs.

To corroborate these preliminary findings on a real dataset, we applied the entire pipeline to the Ashkenazi son HG002 (NA24385) from the Genome in a Bottle (GIaB) project, a well-characterized WGS genome frequently employed for benchmarking variant detection tools (see Supplementary S3.3.2). Similar to the synthetic benchmarking, Hap.py categorized each variant as a TP, FP, or FN and calculated precision, recall, and F1 scores separately for SNPs and INDELs. Importantly, the GeNePi pipeline exhibited a high performance in the detection of SNVs/INDELs also on the real dataset, with F1, recall, and precision metrics in the range of 0.98–0.99 (the specific values and metrics are presented in detail in Table 1).

**Table 1:** Accuracy metrics for SNV, INDEL, and SV benchmarking in the Ashkenazi son HG002 (NA24385) dataset: metrics are reported for each variant caller and for the consensus call set before and after Duphold-based filtering; genome-wide precision, recall, F1 score, and counts of true positives (TP), false negatives (FN), and false positives (FP) are shown.

Software	Type	Precision	Recall	F1	TP	FN	FP
Haplotypecaller	SNP	0.991	0.993	0.992	3 342 594	24 856	30 672
Haplotypecaller	INDEL	0.994	0.993	0.994	555 278	3745	3426
Breakdancer	SV	0.048	0.078	0.059	750	8896	14 923
Lumpy	SV	0.860	0.233	0.366	2244	7402	364
Manta	SV	0.883	0.440	0.587	4243	5403	563
CNVnator	SV	0.312	0.046	0.080	444	9202	978
Consensus	SV	0.908	0.378	0.534	3649	5997	372
Cons. Filt.	SV	0.948	0.368	0.531	3554	6092	196

These results are consistent with previous findings reported in the literature [47], thus supporting the reliability, the accuracy and the robustness of the pipeline. In parallel, for the SVs benchmarking, we utilized the version 0.6 of the Tier 1 dataset, which includes isolated, sequence-resolved deletions and insertions. The benchmarking was performed using Truvari (see Section “Synthetic and real next-generation sequencing data”). The benchmarking was performed on two sets of SVs: the first set was genotyped with Duphold and unfiltered, and the second set was filtered after Duphold.

We assessed the performance of GeNePi in identifying SVs on this dataset. The results showed that the precision (above 0.90 before and after the filtration) is increased compared with the single tools (Table 1) and the total number of FP is significantly decreased compared with most of the other tools, particularly after the filtration with Duphold. This guarantees that the variants identified by the tool have a high probability to be TP. Although the recall is improved after the consensus (compared with at least three of the tools when used independently, i.e. Breakdancer, Lumpy, and CNVnator), it is $\sim $0.36–0.37 in both the two conditions tested (consensus with and without Duphold filtration, respectively). This parameter also affects the F1 score. Depending on the research objectives, it may be desirable to optimize metrics other than precision. To enable this flexibility, the consensus strategy can be directly modified through predefined variables (see Supplementary S2.2.4) and the variants called by each tool are stored to allow further analysis. For instance, adopting the union of all SVs identified by the four tools increases the number of TP, but also raises the FP rate.

We also obtained comparable performance in the detection of SNVs, INDELs, and SVs when analyzing WGS data from human tumor cell lines of the Cancer Cell Line Encyclopedia [48–50], thus supporting the generalizability and robustness of the pipeline we developed (see details in Supplementary S3.3.4).

Additionally, to challenge the acceleration of the pipeline, we used a real-world case scenario on data produced by our high-throughput sequencing facility which can generate about 22–25 genomes (at 30–40$\times $) from a single sequencing run on the S4 flow cell using the Illumina NovaSeq 6000 platform. The pipeline completed the analysis of 22 genomes with an average depth of $30\times $–$40\times $ in $11$ h and $45$ min, with an average execution time for the **PB_germ** module of $53$min. Considering that the NovaSeq 6000 can sequence two flow cells in parallel and that the sequencing takes $\sim 44$ h, we are able to sustain a fully operational workflow using the NovaSeq 6000. We are currently using GeNePi for most of the genomes sequenced in our laboratory.

### Discussion & Conclusion

In recent years, we have witnessed an exponential growth in the production of genomic data due to advances in NGS sequencing technology. This has imposed a concomitant advancement in the computational infrastructure and data processing algorithms. This complexity has prompted the scientific community to develop automated pipelines with the dual objective of standardizing results and optimizing the use of computational resources. In this study, we present GeNePi, a pipeline for short pair-end reads WGS analysis that can be easily deployed into an HPC infrastructure. This pipeline is able to identify and prioritize SNVs, INDELs, CNAs, and SVs. We adopted GPU-accelerated algorithms for the most computationally intensive steps and a configurable filtering process allowing a predefined prioritization of variants to assist researchers in identifying clinically relevant information. The Parabricks implementation of HaplotypeCaller was adopted for the identification of SNVs/INDELs and a consensus of four variant callers for the identification of SVs. The pipeline is designed to be utilized by a team of researchers facilitating the standardization of results and the collaborative use of the annotation databases, while minimizing disk space requirements. We tested the efficacy of our pipeline on two synthetic datasets and on the publicly available sample NA2438 form the GIAB project. This analysis revealed the ability of the pipeline to accurately identify SNVs and INDELs. Notably, the performance of the pipeline was comparable with state-of-the-art tools in the identification of SVs using pair-end short reads, a particularly challenging task in genomic studies.

Compared with other available pipelines [2, 3, 34], the acceleration on GPUs guarantees a faster execution than other free software and allows a competitive execution time with commercial products without additional costs. The GPU-based pipeline demonstrated an average acceleration in execution time ranging from 10 to 78 times compared with CPU-based pipelines, depending on the hardware configuration tested (see in Fig. 2 and Supplementary Fig. S11). This allows us to analyze 22 genomes in <12 total hours, less than the time required to process a single sample using the equivalent CPU-based tools. This result indicates that this method can significantly improve the efficiency of genomic analysis, compared with the GATK alignment and variant calling workflow. The execution time of the germline pipeline we developed on the NVIDIA Clara Parabricks suite (v4.3.0) is comparable to that of the FPGA-based Dragen, which is one of the most commonly used platform for the analysis of large WGS data also in the clinical setting. We also show how the advancements in GPU architecture and algorithm optimization have significantly enhanced performance. We can envision that next-generation GPU will further decrease the computational time (see Parabricks web page). Importantly, the GPU-based computational infrastructure we implemented in our center, which is currently dedicated to the analysis of genomic data, offers the distinct advantage over the FPGA-based Dragen suite of being applicable also to a broader range of research, clinical, and mathematical studies, including multi-omics and radiomic analyses. On a broader scale, while the diffusion of FPGAs is still limited to specific applications the adoption of GPUs is ubiquitous across all fields of data analysis, machine learning and AI, ensuring their accessibility to all institutions.

A configurable filtering process allows a predefined prioritization of the variants to assist researchers in identifying clinically relevant variants. The default configuration of the current version of the pipeline is optimized for the identification of germline mutations, with a focus in identifying non-common variants that may have clinical relevance. Therefore, the default filtration may prove to be overly restrictive in genomic studies where common variants are relevant. Nevertheless, the flexibility offered by Nextflow allows to re-define some of the analysis steps to better align with the specific needs of the laboratory or research project, without rewriting the entire pipeline. In light of the significance of somatic mutations in pathological contexts, such as cancer, future endeavors aim to expand the scope to encompass tissue-specific alterations.

Currently, WGS analysis has been mainly adopted in a research to study particular complex clinical cases, but it is foreseeable that within the next few years, we will assist in the rapid implementation of WGS in the clinical setting [36]. Therefore, it is imperative to establish the necessary infrastructure and educate the personnel to adequately address the challenges posed by this technology. The adoption of GPU acceleration across different fields has significantly catalyzed the rapid advancement of this technology that is now widely available in almost all computational infrastructures. The field of bioinformatics can take advantage of this rapid development by adopting compatible algorithms. The automated pipeline GeNePi that we are proposing exploits the acceleration afforded by the GPU adoption to address some of the challenges posed by NGS. The pipeline enables efficient and reproducible analysis of high-throughput data using standardized data processing and automated prioritization of variants. Based on containerized software, GeNePi could be easily deployed in an HPC infrastructure to support the bioinformatic analyses of a diverse user base comprising students, professionals, and clinicians.

**Key Points**

- We report on GeNePi, a novel, modular bioinformatics pipeline built on the Nextflow framework, designed for efficient and accurate analysis of whole-genome sequencing (WGS) short paired-end reads.
- GeNePi is optimized for high-performance computing environments and relies on graphics processing unit-accelerated algorithms, thus allowing a faster execution compared with either free software or commercial products (without additional costs).
- GeNePi supports various workflows and automates the detection as well as the prioritization of single nucleotide variants, insertions/deletions, copy number variants, and structural variants from raw WGS data.
- Tested on public data, GeNePi demonstrates comparable performances to state-of-the-art tools. When tested on data produced in-house via a high-throughput sequencing facility, GeNePi completes the analysis of 22 genomes (30$\times $) in <12 h.
- GeNePi’s flexible design allows adaptation to various research and clinical settings, significantly enhancing the speed of large-scale genomic analyses, hence boosting the development of National Centers for Computational and Precision Medicine.

## Supplementary Material

GeNePi_SupplInfo_rev_bbag001

## Data Availability

The GeNePi pipeline’s source code is publicly accessible through the GeNePi project page on the IIT GitLab platform: https://gitlab.iit.it/Stefano.Marangoni/genepi. WGS data used for variant detection benchmarking are available from the NIST - Genome In A Bottle Consortium for the Ashkenazim Son (HG002) sample, and from the Sequence Read Archive (SRA) for the two cell lines under accession numbers SRR8670730 (PANC1005) and SRR8670746 (SCLC21H). The reference calls used to perform the benchmarks are available from the SV reference calls v0.6 and SNV reference calls v4.2.1 for the Ashkenazim Son (HG002) sample and on the DepMap Portal pages of PANC1005 and SCLC21H cell lines.
