# Supplementary material for: GeNePi: a graphics processing unit enhanced next-generation bioinformatics pipeline for whole-genome sequencing analysis
Source: Brief Bioinform. 2026 Jan 25;27(1):bbag001. doi: 10.1093/bib/bbag001 (PMC12832024; doi:10.1093/bib/bbag001)
Supplement: GeNePi_SupplInfo_rev_bbag001 [file genepi_supplinfo_rev_bbag001.pdf]

GeNePi Alternative Workflows

GeNePi is based on the Nextflow platform and we adopted Singularity (or equivalently Apptainer) for the containerization of the tools. Consequently, we require that these two softwares and *Make* are already installed. The source code of our pipeline is available for download from the Git repository GeNePi. In the main folder of the project a file *Make* is provided to assist users in the installation process. The *Makefile* is responsible for building the necessary containers in the sub-folder *'bin/def/'* and downloading the required databases (i.e. reference genome, annotation databases) in the sub-folder *'bin/resource/'*. Users interested in a specific sub-workflow can limit the download to a subset of databases by using the dedicated options provided in the Makefile. A complete list of available options is documented in the project's README. The total amount of space required, including containers and database, is approximately 210GB. This step is required only the first time and the user that executes the command must have access on internet (to download the required tools and databases) and the rights to build containers. The time required to accomplish this initial step could be substantial, due to the large amount of data to be downloaded. However, once installed, multiple users can concurrently access and utilize it, without encountering conflicts in their respective executions. It is important to note that the execution of the pipeline does not necessitate internet access. This facilitates the establishment of an isolated perimeter, thereby ensuring the protection of genomic data.

The typical command for running GeNePi is:

```
nextflow run genepi.nf --samples samples_ids --outdir path_to_results
```

As described in the main text this workflow executes all the necessary steps to identify disease-causing variants including single nucleotide variants (SNVs), small insertions or deletions (INDELs), copy number variants (CNVs) and structural variants (SVs) starting from FASTQ files. Alternatively, it is possible to execute only a sub-workflow with the "-entry" option depending on the necessity of the user. This functionality is relevant in case of samples reanalysis.

```
nextflow run genepi.nf -entry wf_name --samples samples_ids --outdir path_to_results
```

Since we considered the deployment on an HPC infrastructure, we adopted *pbspro* as the executor (see Nextflow documentation), however, the Nextflow syntax grants an easy adaptation to other job schedulers. Currently the various workflows have been tested also on *local* executor. It is recommended an initial benchmark by an expert user to optimize the resource allocation and adapt the parameters of the processes to circumvent superfluous resource requests by inexperienced users. The user could produce a report of the execution using the built-in functionalities of Nextflow specifying the parameter *-with-report* or *-with-timeline* (for more details see the related Nextflow documentation):

```
nextflow run genepi.nf --samples samples_ids --outdir path_to_results -with-report my_report.html -with-timeline my_timeline.html
```

In the following paragraph, the principal sub-workflows are described.

- **PB_germ**: this sub-workflow carries out read alignment to the reference genome and the simultaneous call of single-nucleotide variants (SNVs) and small insertions/deletions (INDELs) using the GPUs-accelerated Parabricks Germline pipeline. Additionally, it includes a variant quality check through GATK VariantFiltration (further details on the criteria adopted in 2.1) and the coverage estimation by samtools (v1.21). It is important to note that the variant quality assessment does not discard low-qualitity variants; rather, it functions by providing additional information in the filter field of the VCF, including the designation 'PASS' or the names of unsuccessful filters.

Fig. S1. Schematic representation of the PB_germ sub-workflow.

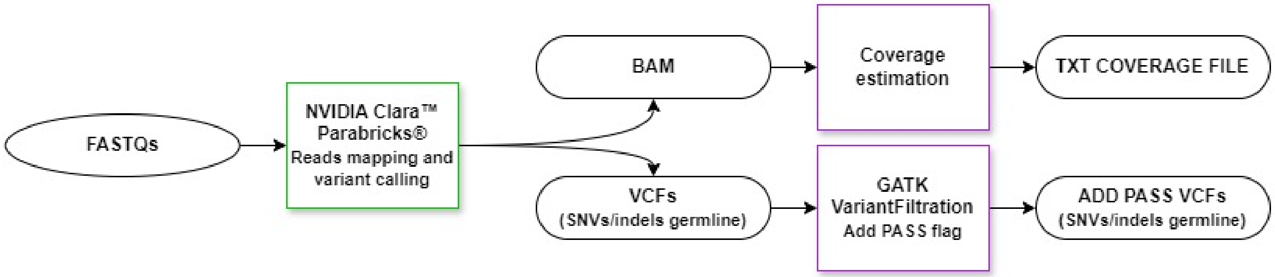

- **SNV_annot**: this sub-workflow adds biological information to the variants in each VCF. Initially, the multi-allelic variants present in the VCF are separated into discrete rows, using Bcftools (v1.21.0). Subsequent to these preparatory steps, the VCF is annotated with three annotation tools applied sequentially: SnpEff (v5.1) [3] to include information on the transcripts affected by the variant and the putative effect on the protein or on the transcript; ANNOVAR (databases updated to 23/01/2024) [4] to include information on the frequency of the variant in the population studies, score predictions, predicted effects of pathogenicity or clinically observed effects; and COSMIC (Catalogue of Somatic Mutations in Cancer) a database reporting the somatic mutations in human cancer [5]. As this annotation is primarily relevant to cancer studies, the user is permitted to bypass this step by setting the flag *COSMIC_FLAG* to false.

Fig. S2. Schematic representation of the SNV_annot sub-workflow.

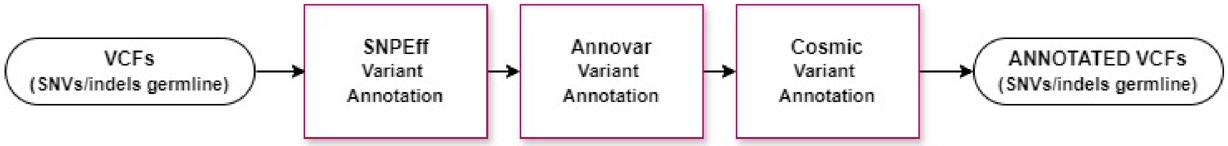

- **SNV_filt**: this sub-workflow takes in input a list of annotated VCF files and prioritizes potentially disease-causing variants based on the biological information available in the annotation. The prioritization scheme is represented by multi-stage filtration, the function of which is to refine the variant selection by excluding those that do not meet the increasingly restrictive criteria established over the five steps:

  1. Rare variants in the reference genome (GnomAD v4.0) filtration;
  2. Relevant variants according SNPEff/Annovar Annotation;
  3. PASS variants filtration;
  4. Gene list based variants filtration;
  5. Pathogenic variants filtration.

  A complete description of each step is reported later in the text (see 2.2.2). This module allows a drastic reduction of the variants that should be manually checked by the researchers. Although this could be an advantage when the research is focused on clinically relevant variants on a specific gene panel, some analyses do not have a predefined set of relevant genes nor are focus on variants with predicted pathogenic effects. Therefore, we allow the user to skip the last two step of the filtration using the parameter *FILT_PANEL_FLAG*.
- **CNVkit_wf**: this sub-workflow is based on the CNVkit copy number caller pipeline and handles the detection and visualization of large CNVs starting from BAM files. For this workflow to works properly it is required that the user generate a reference following the instructions on the CNVkit website.

Fig. S3. Schematic representation of the CNVkit_wf sub-workflow.

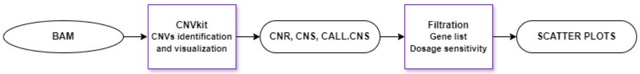

- **SV_consensus**: it conducts the SVs calling, consensus, annotation and filtering processes starting from BAM files. Initially, SVs are called by 4 callers: Manta, Lumpy, Breakdancer and CNVnator. Then it executes the annotation workflow with SVAFotate [10] and AnnotSV [11] and finally the in-house filtering workflow.

Fig. S4. Schematic representation of the SV_consensus sub-workflow.

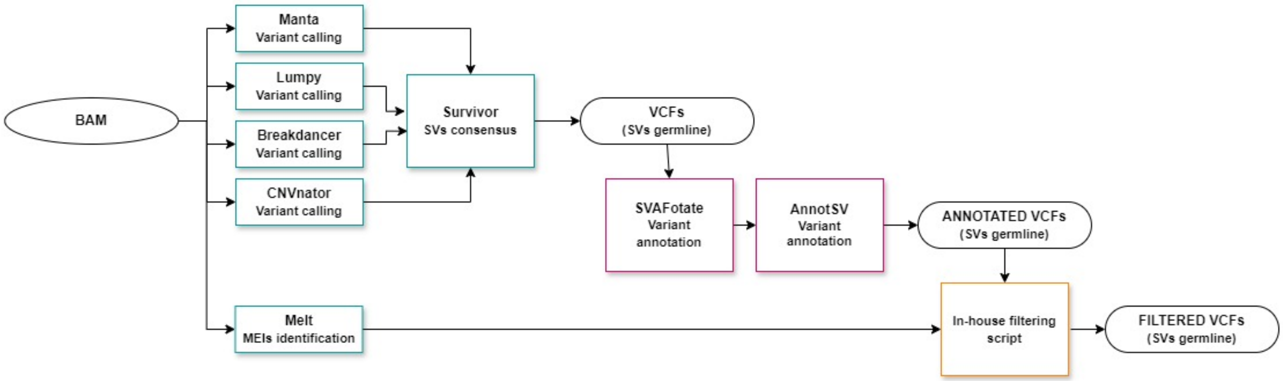

- **SV_annot_filt**: it executes the SVs annotation and filtering processes starting from the consensus VCF file.

Fig. S5. Schematic representation of the SV_annot_filt sub-workflow.

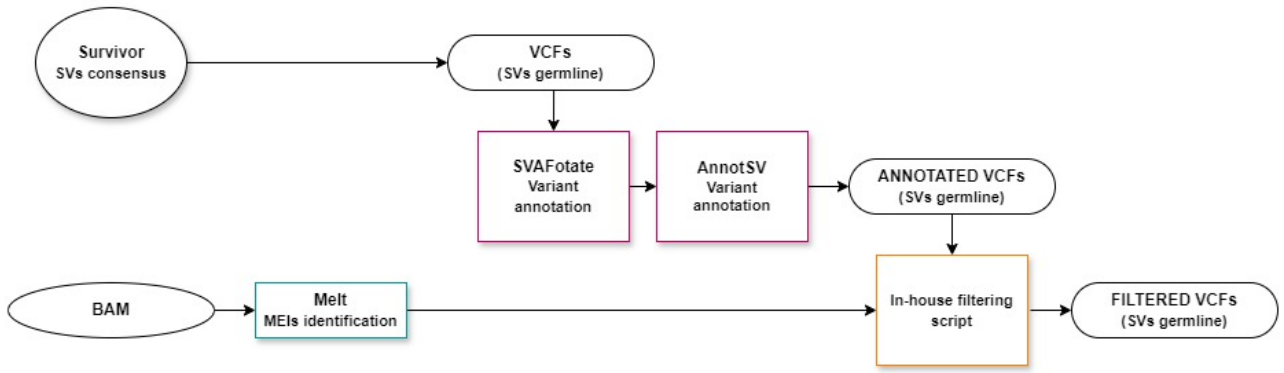

- **PB_Germ_SV**: it carries out read alignment to the reference genome and the single nucleotide variant (SNV) calling using the GPUs-accelerated Parabricks Germline pipeline, followed by the SVs identification, consensus, annotation and filtering processes starting from BAM files.

Fig. S6. Schematic representation of the PB_Germ_SV sub-workflow.

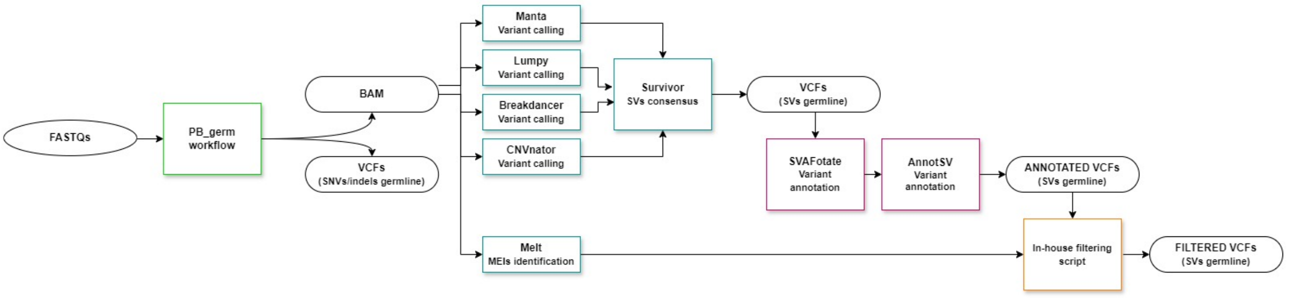

- **SNV_AddPass_annot_filt**: this sub-workflow, starting from the VCF file, executes the SNVs annotation and filtering processes.

Fig. S7. Schematic representation of the SNV_AddPass_annot_filt sub-workflow.

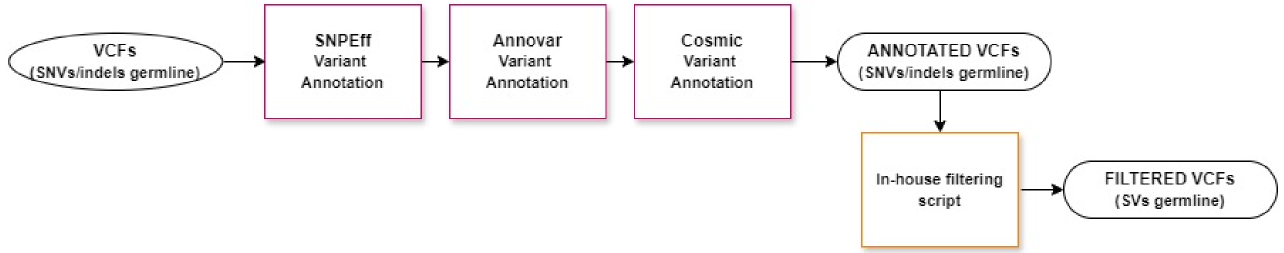

- **PB_Germ_SNV**: it performs reads alignment to the reference genome and the single nucleotide variant (SNV) calling using the GPU-accelerated Parabricks Germline pipeline, followed by the annotation and filtering processes.

Fig. S8. Schematic representation of the PB_Germ_SNV sub-workflow.

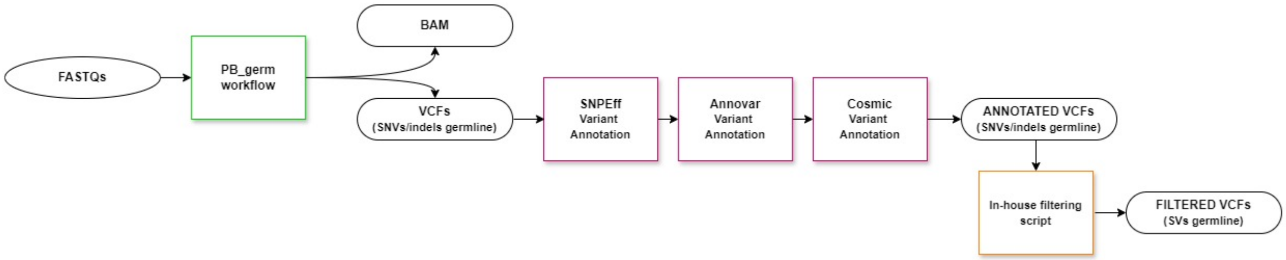

- **GenerateReport**: this sub-workflow collects metrics and statistics from the execution of one or multiple workflows and uses these informations to generate a report using MultiQC. If launched independently as a stand-alone workflow, the workflow searches in the defined output folder and collects metrics already available and computes new metrics as the VCF statistics using *bcftools stats*. The final report will include metrics on duplication metrics, base quality score recalibration, BAM statistics (computed using the functions *stats* and *coverage* from the *samtools* toolkit), and prioritized variants metrics at different filtering steps (see Fig. S9).

Fig. S9. Example of a MultiQC report produced by the GenerateReport sub-workflow.

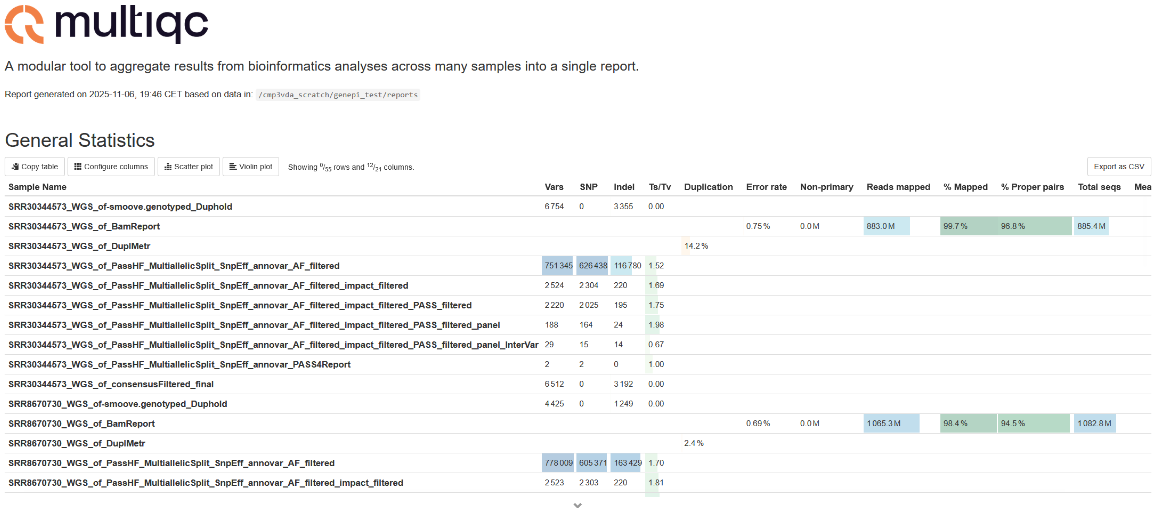

multiqc

A modular tool to aggregate results from bioinformatics analyses across many samples into a single report.

Report generated on 2025-11-06, 19:46 CET based on data in: `/cmp3vda_scratch/genepi_test/reports`

General Statistics

Copy table | Configure columns | Scatter plot | Violin plot | Showing 0/55 rows and 12/21 columns. | Export as CSV

Sample Name	Vars	SNP	Indel	Ts/Tv	Duplication	Error rate	Non-primary	Reads mapped	% Mapped	% Proper pairs	Total seqs	Mea
SRR30344573_WGS_of-smoove.genotyped_Duphold	6 754	0	3 355	0.00								
SRR30344573_WGS_of_BamReport						0.75 %	0.0 M	883.0 M	99.7 %	96.8 %	885.4 M	
SRR30344573_WGS_of_DuplMetr					14.2 %							
SRR30344573_WGS_of_PassHF_MultiallelicSplit_SnpEff_annovar_AF_filtered	751 345	626 438	116 780	1.52								
SRR30344573_WGS_of_PassHF_MultiallelicSplit_SnpEff_annovar_AF_filtered_impact_filtered	2 524	2 304	220	1.69								
SRR30344573_WGS_of_PassHF_MultiallelicSplit_SnpEff_annovar_AF_filtered_impact_filtered_PASS_filtered	2 220	2 025	195	1.75								
SRR30344573_WGS_of_PassHF_MultiallelicSplit_SnpEff_annovar_AF_filtered_impact_filtered_PASS_filtered_panel	188	164	24	1.98								
SRR30344573_WGS_of_PassHF_MultiallelicSplit_SnpEff_annovar_AF_filtered_impact_filtered_PASS_filtered_panel_InterVar	29	15	14	0.67								
SRR30344573_WGS_of_PassHF_MultiallelicSplit_SnpEff_annovar_PASS4Report	2	2	0	1.00								
SRR30344573_WGS_of_consensusFiltered_final	6 512	0	3 192	0.00								
SRR8670730_WGS_of-smoove.genotyped_Duphold	4 425	0	1 249	0.00								
SRR8670730_WGS_of_BamReport						0.69 %	0.0 M	1 065.3 M	98.4 %	94.5 %	1 082.8 M	
SRR8670730_WGS_of_DuplMetr					2.4 %							
SRR8670730_WGS_of_PassHF_MultiallelicSplit_SnpEff_annovar_AF_filtered	778 009	605 371	163 429	1.70								
SRR8670730_WGS_of_PassHF_MultiallelicSplit_SnpEff_annovar_AF_filtered_impact_filtered	2 523	2 303	220	1.81								

In addition to these workflows, the following ones are also available. The description is available on the Git repository: **PB_Germ_SNV_nofilt** ; **PB_Germ_SNV_CNVkit** ; **PB_Germ_SNV_nofilt_SV** ; **PB_Germ_SNV_nofilt_CNVkit_SV** ; **PB_Germ_CNVkit_SV** ; **Coverage_wf** ; **AddPassHardFilter_wf** ; **SNV_annot_filt** ; **SNV_annot** ; **SNV_filt** ; **Melt_wf**.

GeNePi Supplementary Details

Soft-Filtration

The VCF generated by the variant calling process is subsequently soft-filtered using the tool VariantFiltration (GATK). Variants that pass all the quality filters report the value "PASS" on the FILTER column, while for the others, the list of failed filters is reported. The list of filtering condition is:

- $QD < qd\_value$ filter-name *QD2* ;
- $QUAL < qual\_value$ filter-name *QUAL30* ;
- $SOR > sor\_value$ filter-name *SOR3* ;
- $FS > fs\_value$ filter-name *FS60* ;
- $MQ < mq\_value$ filter-name *MQ40* ;
- $MQRankSum < mqranksum\_value$ filter-name *MQRankSum-12.5* ;
- $ReadPosRankSum < readposranksum\_value$ filter-name *ReadPosRankSum-8*

In the current version, the parameters are hardcoded and the following values are adopted: $qd\_value = 2$, $qual\_value = 30$, $sor\_value = 3$, $fs\_value = 60$, $mq\_value = 40$, $mqranksum\_value = -12.5$ and $readposranksum\_value = -8$.

Filtering and Annotation

SNVs/INDELs Annotation

The annotation is based on three steps:

- **SnpEff annotation**: SnpEff introduces information on the effect prediction of each genetic variants in known genes (such as amino acid changes, impact, loss of function). It is possible to redefine the folder where the output VCF is published using the parameter *SNPEFF_FOLDER* (default *01_SnpEff*)
- **Annovar annotation**: annotates functional consequences of genetic variation and include information from external databases (e.i. gnomAD, ClinVar) or previously developed mutation prediction algorithms (e.i. CADD, SIFT, Polyphen).
- **COSMIC annotation**: the annotation with COSMIC adds in the field ID of the VCF the Catalogue of Somatic Mutations in Cancer (COSMIC) identifier and could be used to identify the variants in this important database of oncological mutations. The annotation is based on a VCF file, *CosmicCodingMuts.vcf.gz*, that requires to be downloaded manually by the user to access the COSMIC database. Since this information is specific to the oncological pathology, in the current version is not included in the subsequent filtering steps, but it could represent an additional source of information for a final manual curation. It is possible to modify the folder where the output VCF is published, using the parameter *COSMIC_FOLDER* (default *02_cosmic_annotation*)

SNV/INDEL Filtration

The multi-step filtration process is designed to progressively eliminate variants from the preceding step and store the filtered VCFs in a dedicated folder. The steps of the filtration process are listed below:

- **Filtering on the variant frequency in the population**: in this first step, common polymorphisms are filtered out using SnpSift with a double threshold. The parameters that can be set relatively to this filtration are the following. The folder where the results are published, *FILTER_AF_FOLDER* (default *03_filter_af*); the population used to compare the frequencies, *AF* (default *gnomad40_genome_AF*), notice that this parameter should be correctly set depending on the Annovar annotation; the frequency ranges identified by the two parameters *AF_VALUE* (default 0.05) and *AF_NVALUE* (default 0.95).
  NOTE: all the variants with frequency *af* greater than *AF_VALUE* and smaller than *AF_NVALUE* are discarded.
- **Filtering on the effect on protein structure**: in the second step, we searched variants with the potential to impact protein structure and splicing or with known clinical effects with SnpSift, classified with "HIGH" or "MODERATE" impact according to SnpEff, or those with pathogenic effects as defined by Clinvar (the file containing the list of accepted values is defined by the parameter *set_Clinvar*) or InterVar [7] (*set_Intervar*), or those affecting splicing sites with a dbscSNV prediction scores above 0.6 or with a CADD score higher than 25. It is possible to specify the folder where the results will be saved, *FILTER_IMPACT_FOLDER* (default *04_filter_impact*), the RF and ADA value for splicing site and the CADD value respectively with the parameters *snv_rf_score*, *snv_ada_score* and *cadd_phred*.
- **Filtering low quality variants**: in the third step, we select variants that meet a minimum quality standard, as defined by the FILTER fields of the VCF. This step is crucial to minimize false positives in the final VCF. It is possible to specify the folder where the results will be saved, *FILTER_PASS_FOLDER* (default *05_filter_pass*).
- **Filtering on a selected gene list**: In the forth step, variants are filtered based on a gene list, specified using the parameter *PANEL_GENE_FILE*. By default, the pre-configured set of genes has been adapted for oncology and the list contains genes present in the Cancer Gene Census (CGC) Project [8] as well as in the cancer panels of Genomics England PanelApp. It is possible to specify the folder where the results will be saved, *PANEL_FOLDER* (default *06_panel_gene_filtration*) and the gene panel file with the parameter *PANEL_GENE_FILE*.
- **Filtering on the expected pathogenicity**: in the final step, we evaluates the pathogenicity scores of different prediction tools, collecting variants classified as pathogenic (or likely pathogenic) for InterVar or Clinvar. Yet, it should be noted that since most of the variants are classified VUS they are thus excluded from this evaluation. To circumvent the exclusion of potentially novel, disease-causing variants previously classified as VUS, an evaluation of the score generated by the CADD algorithm (CADD

>20) is also conducted and a restrictive threshold for the MAF in the GnomAD database (gnomAD <1%) is applied. It is possible to specify the folder where the results will be saved, *INTERVAR_FOLDER* (default *07_InterVar*)

It should be noted that since most of the variants are classified VUS they are thus excluded from this evaluation. To circumvent the exclusion of potentially novel, disease-causing variants previously classified as VUS, an evaluation of the score generated by the CADD algorithm (CADD >20) is also conducted and a restrictive threshold for the MAF in the GnomAD database (gnomAD <1%) is applied. In the event that no disease-causing variants are detected in the initial analysis, re-evaluation may be necessary. To this end, we retained the VCF produced at each step of the filtration process, enabling expeditious retrieval of previously discarded variants.

CNVkit

The CNVs analysis is performed using a pre-built reference file with a window of 5kbp, which the user have to produce by him self (follow the instructions in the CNVkit documentation) and given through the *CNVKIT_COVERAGE_REFERENCE* parameter. The outputs of the CNVkit process are then fed to our script that combines the data to produce a series of scatter plots (one for each chromosome), where deletions and duplications are shown and genes of interest are highlighted. The scatter plots reported the genomic position in megabases (Mb) on the x-axis, and the log2 ratio between the number of sample reads and the number of reference reads on the y-axis. Local coverage (cnr file) is reported with a gray dot, while the continuous segment are reported in cyan (cns file). Deletions (blue) and duplications (red) are highlighted on the basis of the evidence collected in the call.cns file. The centromere regions are marked in yellow and difficult to map regions are marked in purple.

SV consesus

The module **sv_consensus** implements a 4 tools consesus in which SVs are grouped based on the SV type and, for each group, a consensus is run using Survivor (v1.0.7) [9]. The default sets the minimum number of supporting callers at 2 and 1000bp is established as the maximum distance between breakpoints. These parameters that define the consensus strategy could be modified using the parameters: *breakpoint_dist*, *min_tool_calls*, *use_type*, *use_strand*, *dist_based*, *params.min_sv_size*. For example, to consider the union of all the variants detected by the four SV callers, it is possible to set *min_tool_calls*=1. After Survivor, variants are genotyped with Smoove and analyzed with Duphold. Consequently, merged SV calls are annotated with SVAFotate (v0.0.1) [10] and AnnotSV (v3.2.3) [11]. For the SVAFotate process, the population SV source data is given with the parameter *BED_SVAFotate*, by default SVAFotate_core_SV_popAFs.GRCh38.v4.1.bed.gz, the minimum Overlap Fraction threshold is given *OVERLAP_FRACTION*, by default "0.5", and the extra annotation is given with the parameter *ANNOTATIONS*, by default set to "best". On parallel to the 4 callers, a workflow for the tool MELT [12] detects the mobile element insertion, when the parameter *MELT_FLAG* is set on "true" by default. The same annotation process is done also on the VCF obtained with MELT.

SV filtration

The module **sv_filt** uses an in-house filtering Python script to apply the required filtration and produce a table in Excel format to prioritize variants. The script accepts as input: the two TSV file generated by AnnotSV, from the consensus call and from MELT; the BAM file associated with the TSVs; the name of the output; a BED file containing a list of genes of interest and used to filter the variants given with the parameter *BED_for_filtering*; a flag used to optionally discard all the variants with a population frequency above 1% ($MAF < 1\%$); the dosage sensitivity map from the article [1] given as a TSV file with the parameter *TSV_DOSAGE*. Since the execution of MELT is optional the associated TSV is also optional but if provided, the output includes a second table with a consensus between the calls of Manta and MELT. Once the duplication and the deletion are prioritized, the script uses also samplot [2] to generate plot of the SV using the information contained in the BAM file. These plots could help the researcher in manually cured false positive identification.

GeNePi Benchmark

Infrastructure setting

Most of our tests have been performed on an HPC infrastructure with two Front-End (FE) nodes and twelve computational nodes. The FE nodes were identical and equipped with two processors Intel Xeon 4214 and 96GB RAM. The computational nodes are accessible from the FE nodes via the job scheduler OpenPBS and they are grouped into three queues: i) an eight-node queue each with two CPUs, Intel Xeon 6242, 192GB RAM, two 500GB HDDs connected via a 10GBE port (we will refer to this hardware configuration as CPU nodes); ii) a queue for analyses on Nvidia V100 architecture composed by two identical nodes each equipped with two Xeon6244, 384GB RAM, two 500GB HDDs, and two NVIDIA Tesla V100-PCIE-32GB (referred to as 2xV100); iii) a queue for analyses on Nvidia A100 architecture composed by two nodes with different characteristics iii-A) one CPU Intel RX2540 M6, 48 cores, 395GB RAM, and two GPUs Nvidia Ampere A100-PCIE-40GB (referred to as 2xA100) and iii-B) two CPUs AMD GX2460 M1 64 cores and four GPUs NVIDIA A100-PCIE-40GB (referred to as 4xA100). All nodes are connected to the shared storage with Infiniband. Lately thanks to a collaboration with EMBL Heidelberg Data Science Centre team, we had the opportunity to perform some test also on the EMBL Cluster that host nodes equipped with latest GPU architecture: node with 4×V100 GPUs, Dual Xeon 6252 CPUs (24 cores each), 384 GB RAM; nodes with 8×A100 GPUs, two AMD Epyc Milan 7663 CPUs (56 cores each), 1 TB RAM ; node with 8×H100 SXM GPUs, two AMD Epyc Genoa 9654 CPUs (96 cores each), 1.5TB RAM; node with a single Epyc 9754 CPUs, 384 GB RAM. The computational nodes are accessible to the FE nodes by the SLURM job scheduler.

PB_germ Benchmark and CPU equivalent workflow

We adopted the Parabricks germline variant pipeline (v4.3.0) and it is characterized by the raw sequencing paired-end reads alignment to the human reference genome (by default, all database and reference genomes will be referenced to the GRCh38/hg38). The equivalent CPU-based pipeline is described in the Nvidia documentation. Briefly, reads are aligned to the reference genome using the algorithm BWA-Mem and sorted using *samtool* into a BAM file. This file is then processed with MarkDuplicates, BaseQualityScoreRecalibrations and ApplyBQSR in order to reduce base-calling and alignment artifacts. Finally, the resulting BAM file is used to call the variants present in the sample using the tool HaplotypeCaller (GATK). The variants are collected into a Variant Calling Format (VCF) file harboring millions of variants.
The execution time of the workflow **PB_germ** of the GeNePi pipeline across various nodes (CPU, V100x2, A100x2 and A100x4), described in table S1, show an almost linear scaling with the average read depth of the BAM files. This relation is schematically illustrated in Figure S10, providing a comparison of performance in each hardware configuration.

Fig. S10. Pipeline execution time on WGS datasets sequenced at different read depths. The orange (2x Nvidia GPU V100), gray (2x Nvidia GPU A100), and yellow (4x Nvidia GPU A100) curves represent different hardware configurations tested with the GPU-accelerated germline module using Nvidia Clara Parabricks 4.3, while the blue curve corresponds to the CPU-based implementation.

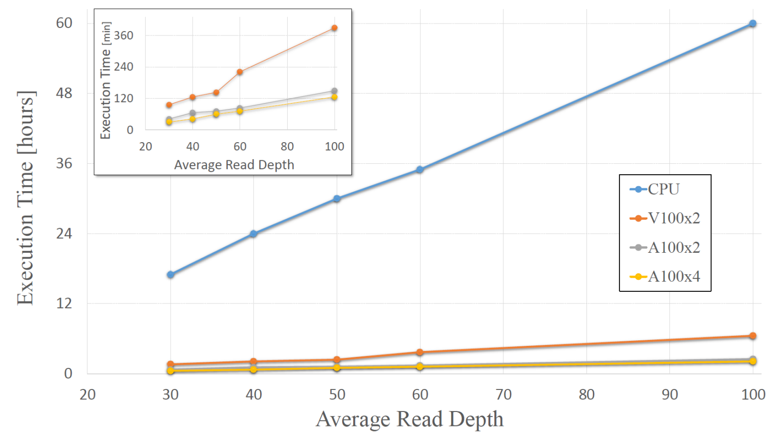

Table S1. Comparison of the execution time of the Nvidia Clara Parabricks germline_pipeline on different hardware and compared to the equivalent pipeline on CPU. We tested the pipeline on WGS human data and we considered different average depths. A complete description of the hardware is available in the section 3.1

Avg. Reads Depth	CPU	GPU-2xV100	GPU-2xA100	GPU-4xA100
30x	17h	1h 36m	42 m	30m
40x	24h	2h 06m	1h 06m	42m
50x	30h	2h 24m	1h 12m	1h 00m
60x	35h	3h 42m	1h 24m	1h 12m
100x	60h	6h 30m	2h 30m	2h 06m

We evaluated the performance of the germline_pipeline sub-workflow on whole-genome sequencing (WGS) data from the HG002 sample (average coverage: 60×) across multiple computational environments (see Fig.S11). Our primary goal was to quantify the impact of different GPU architectures, GPU allocation strategies, and software versions on runtime efficiency. As expected, adopting more recent GPU architectures significantly reduced computational time compared to older hardware. Increasing the number of GPUs per job further improved performance; however, this optimization introduces a trade-off between processivity and throughput. While allocating more GPUs to a single process minimizes runtime for that job, it reduces the ability to run multiple samples concurrently on the same node. Therefore, resource allocation strategies should be tailored to user priorities—whether minimizing turnaround time for individual samples or maximizing overall throughput for batch processing. Beyond hardware considerations, software evolution plays a critical role in performance optimization. Fixing the hardware configuration, we observed that Clara Parabricks version 4.6 reduced runtime by approximately 30% compared to version 4.3. This improvement underscores the rapid pace of development in GPU-accelerated genomics workflows and highlights the importance of maintaining up-to-date software environments to leverage these gains. However, it is worth noting that newer versions may discontinue support for older GPU architectures, such as V100, which can influence deployment decisions in heterogeneous computing environments. Implications for Genomics Workflows These findings confirm a consistent trend: both hardware and software advancements contribute substantially to reducing computational bottlenecks in large-scale genomic analyses. Users must carefully balance hardware allocation, software compatibility, and workflow design to achieve optimal performance aligned with their operational goals. The observed improvements reinforce the transformative potential of GPU acceleration in genomics and the need for continuous benchmarking as technologies evolve.

Fig. S11. Benchmarking the germline sub-workflow on HG002 WGS (60× coverage) using the GPU-accelerated implementation across different GPU architectures and the CPU equivalent. (A) Pipeline runtime (logarithmic scale) under varying GPU allocations and architectures. (B) Speed-up relative to the CPU-based GATK implementation. Newer GPU architectures improve performance at fixed GPU counts, while doubling GPUs introduces diminishing returns, highlighting the trade-off between efficiency and throughput. The latest Parabricks release enhances performance but discontinues support for older GPUs (e.g., V100). Optimal GPU configuration should be guided by user-specific requirements.

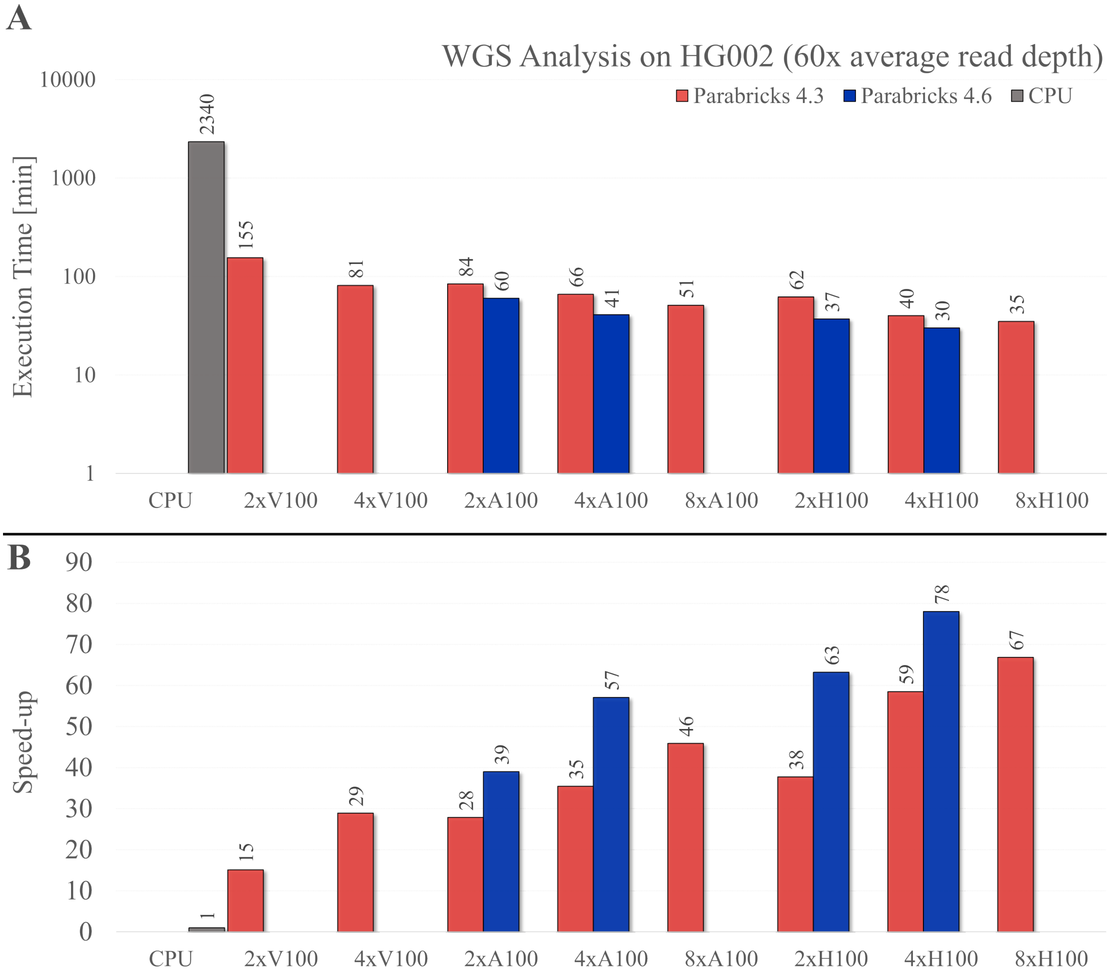

Variants Detection Benchmark

We performed the benchmarking analysis using the variantbenchmarking module of the nf-core pipeline, leveraging Hap.py, a tool specifically designed for small germline variant benchmarking, for the SNV benchmark and Truvari [13], a toolkit for benchmarking, merging, and annotation of SVs, for the SVs benchmark. Each tools classify variants as true positive (TP), false positive (FP), or false negative (FN). Moreover, precision, recall and the F1 score were calculated separately for SNPs and INDELs, by Happy, for SV, by Truvari, to provide a detailed analysis of variant calling performances. Independent counters are used for SNPs and INDELs to provide a detailed analysis of variant calling performances and, additionally, the evaluation is repeated considering only the variants that pass all the quality filter (flag PASS in the field *filter* in the VCF). The counts of TP, FP, FN are then used to compute the precision, recall and F1-score.

$$Precision(P) = \frac{TP}{TP + FP}$$

$$Recall(R) = \frac{TP}{TP + FN}$$

$$F1_{score}(F1) = 2\frac{PR}{P + R}$$

These quantities gave important information on the true positive rate (recall), false positive rate (precision) and false discovery rate ($FDR = 1 - P$).

Generation of the synthetic data

The first synthetic dataset, used to validate the SNP/INDELs calls, was generated using the tool wgsim. The pair-end reads of 150bp each were generated from the reference genome *hg38*.

The truth set contains 2924993 random SNVs/INDELs to serve as the ground truth for benchmarking purposes. One of the issues in using this software is that the resulting truth set is not in a standard VCF format and especially in sequence with repeated nucleotides could create some discrepancy. For example the variant CTT mutated in CTTT is denoted in the truth-set as the insertion of a T at the center of the TT pair (chr1:114766:T-TT) unlikely in the VCF where is still an insertion of a T but between the CT pair chr1:114765:C-CT. These difference in identification, although not relevant for the sequence change, lower the number of INDELs considered as true positives, and increase both false negatives and false positives. For this reason we believe the real precision and recall in the identification of INDELs are underestimated.

The second synthetic dataset was generated using simuG [6], a tool designed for simulating various types of SVs, such as deletions, duplications, insertions, inversions and translocations, as already anticipated in **??**. We generated the Structural Variants depicted in table 3.3.1 following the instructions in the GitHub page.

Table S2. Classes of synthetic structural variants (SVs) simulated with the bioinformatic tool simuG and breakpoints identified by our pipeline.

Synthetic SVs	START	START (consensus)	END	END (consensus)
TRA	chr1:46317066	chr1:46317066	A]chr7:143341553]	N[chr7:143341554][
TRA	chr10:21640100	chr10:21640100	A[chr11:85963122[	N[chr11:85963122[
DEL	chr6:162222411	chr6:162222476	chr6:162470829	chr6:162470830
INV	chr3:44699498	chr3:44699497	chr3:44700793	chr3:44700793

Real data retrieval and preprocess

In order to perform the benchmark of both SNVs and SVs called by the pipeline, we had to retrieve the genome and the "truth sets" for the HG002 dataset. Specifically, the high-coverage BAM file "HG002.GRCh38.60x.1.bam" was used as the source data to be analyzed, and the VCF file "HG002_GRCh38_1_22_v4.2.1_benchmark.vcf.gz" as the ground-truth set for SNVs and INDELs and "HG002_SVs_Tier1_v0.6.vcf.gz" as the ground-truth set for SVs. In order to test the complete workflow, we first converted the BAM file into a pair of FASTQ files using the bam2fastq tool. Then we processed them with our pipeline.

Benchmark results

For the first synthetic dataset, we performed the benchmarking analysis using the variantbenchmarking module of the nf-core pipeline, leveraging Hap.py, a tool specifically designed for small germline variant benchmarking. Results are shown in 3.3.3.

For the second synthetic dataset, we compared the breakpoint positions of the generated SVs with the breakpoints detected. In table 3.3.1 is shown that our pipeline is able to detect SVs with high precision.

Table S3. Accuracy metrics for the benchmarking of SNVs and INDELs in the synthetic dataset. The data are shown for all variants identified by the variant caller, referred as ALL, and for the variants that pass the quality, referred as PASS. The overall accuracy (F1), recall, precision, and counts of true positives (TP), false negatives (FN) and false positives (FP) are shown over the whole genome.

Type	Filter	F1	Recall	Precision	TP	FN	FP
INDEL	ALL	0.738	0.869	0.641	381153	57407	213268
INDEL	PASS	0.717	0.573	0.957	251281	187279	11188
SNP	ALL	0.992	0.985	0.9997	2449279	37130	651
SNP	PASS	0.982	0.964	0.9999	2396801	89608	241

Benchmark on cancer cell lines

To further assess the robustness and generalizability of GeNePi, we analyzed publicly available WGS data from two human cancer cell lines, PANC1005 and SCLC21H, obtained from the Cancer Cell Line Encyclopedia (CCLE) [14]. PANC1005 originates from pancreatic ductal adenocarcinoma, whereas SCLC21H is derived from small cell lung cancer. Illumina WGS data were retrieved from the NCBI Sequence Read Archive under accession numbers SRR8670730 (PANC1005) and SRR8670746 (SCLC21H). Across both datasets, GeNePi detected an average of 4.5 million SNVs/INDELs and approximately 3300 SVs. We compared these results with alterations reported for these cell lines in the NCBI portal (Table 3.3.4). For PANC1005, the pipeline correctly identified 98.8% (84/85) of single-nucleotide variants and small insertions/deletions, while the structural variant (SV) consensus captured 93.5% (29/31) of reported SVs. The single SNV missed by HaplotypeCaller exhibited a low allelic fraction (6/31 reads), a scenario uncommon in germline samples but frequent in tumor samples with subclonal populations. On the highly rearranged SCLC21H genome, GeNePi detected 95.5% (468/490) of SNVs and INDELs. Although the SV consensus module was originally designed for germline samples, the pipeline identified 75.0% (60/80) of structural variants and, leveraging the pipeline's modularity, we further detected six compound heterozygous deletion/duplication events during pre-filtering, increasing SV sensitivity to 83%. Additionally, the massive alterations detected on chromosome 8 (more than 300) are consistent with the known chromothripsis event [15].

The SNV_filt module prioritized approximately 23 variants per sample. In PANC1005, prioritization included key oncogenic alterations such as the well known activating mutation KRAS:p.G12D and the loss of function TP53:p.I255N. In SCLC21H, the 27 prioritized variants included the RB1 stop-gain mutation (p.S829X), the TP53 missense variant (p.P278R), and other relevant changes such as APC (p.Ala766Ser) and PALB2 (p.E657X).

Although GeNePi was originally developed for germline analysis, its flexibility and modularity enable adaptation to other WGS datasets, including cancer genomes with complex rearrangements.

Table S4. Benchmarking of GeNePi in the detection of SNVs, INDELs and SVs in the cell lines PANC1005 (SRR8670730) and SCLC21H (SRR8670746). We assume as truth set the variants reported in the DepMap portal.

Cell Lines	SNV-TruthSet	Calls	SNV-detected	Recall	Prioritized	SV-TruthSet	Calls	SV-detected	Sensitivity
PANC1005	85	4510957	84	0.988	19	31	3301	29	0.94
SCLC21H	490	4540187	468	0.955	27	80	3522	60(+6)	0.75(0.83)

References

1. Collins, Ryan L., et al. "A cross-disorder dosage sensitivity map of the human genome." *Cell* 185.16 (2022): 3041-3055.
2. Belyeu, Jonathan R., et al. "Samplot: a platform for structural variant visual validation and automated filtering." *Genome biology*, 161.22 (2021).
3. Cingolani, P., et al. "A program for annotating and predicting the effects of single nucleotide polymorphisms, SnpEff: SNPs in the genome of Drosophila melanogaster strain w1118; iso-2; iso-3" *Fly*, 6 (2012): 80-92.
4. Wang, Kai, et al. "ANNOVAR: functional annotation of genetic variants from high-throughput sequencing data" *Nucleic acids research*, 38.16 (2010): 164-164.
5. Tate, John G., et al. "COSMIC: the catalogue of somatic mutations in cancer" *Nucleic acids research*, 47.D1 (2019): D941-D947.
6. Yue, Jia-Xing, et al. "simuG: a general-purpose genome simulator" *Bioinformatics* 35.21 (2019): 4442-4444.
7. Li, Quan, et al. "InterVar: clinical interpretation of genetic variants by the 2015 ACMG-AMP guidelines" *The American Journal of Human Genetics* 100.2 (2017): 267-280.

8. Sondka, Zbyslaw, et al. "The COSMIC Cancer Gene Census: describing genetic dysfunction across all human cancers" *Nature Reviews Cancer*, 18.11 (2018): 696-705.
9. Jeffares, DC, et al. "Transient structural variations have strong effects on quantitative traits and reproductive isolation in fission yeast." *Nature Communacation* 8 (2017): 14061.
10. Nicholas, Thomas J., et al. "Annotation of structural variants with reported allele frequencies and related metrics from multiple datasets using SVAFotate" *BMC bioinformatics* 23.1 (2022): 490.
11. Geoffroy, Veronique, et al."AnnotSV: an integrated tool for structural variations annotation" *Bioinformatics* 34.20 (2018):3572-3574.
12. Gardner, Eugene J., et al. "The Mobile Element Locator Tool (MELT): population-scale mobile element discovery and biology" *Genome research* 27.11 (2017):1916-1929.
13. English, Adam C., et al. "Truvari: refined structural variant comparison preserves allelic diversity" *Genome Biology* 23.1 (2022): 271.
14. Bepler, Gerold, et al. "Markers and characteristics of human SCLC cell lines: neuroendocrine markers, classical tumor markers, and chromosomal characteristics of permanent human small cell lung cancer cell lines." *Journal of cancer research and clinical oncology* 113.3 (1987): 253-259.
15. Stephens, Philip J., et al. "Massive genomic rearrangement acquired in a single catastrophic event during cancer development." cell 144.1 (2011): 27-40.
